# Steroid Metabolome Analysis in Disorders of Adrenal Steroid Biosynthesis and Metabolism

**DOI:** 10.1210/er.2018-00262

**Published:** 2019-07-11

**Authors:** Karl-Heinz Storbeck, Lina Schiffer, Elizabeth S Baranowski, Vasileios Chortis, Alessandro Prete, Lise Barnard, Lorna C Gilligan, Angela E Taylor, Jan Idkowiak, Wiebke Arlt, Cedric H L Shackleton

**Affiliations:** 1 Department of Biochemistry, Stellenbosch University, Stellenbosch, South Africa; 2 Institute of Metabolism and Systems Research, University of Birmingham, Birmingham, United Kingdom; 3 Centre for Endocrinology, Diabetes and Metabolism, Birmingham Health Partners, Birmingham, United Kingdom; 4 Department of Paediatric Endocrinology and Diabetes, Birmingham Women’s and Children’s Hospital NHS Foundation Trust, Birmingham, United Kingdom; 5 Department of Endocrinology, Queen Elizabeth Hospital, University Hospitals Birmingham NHS Foundation Trust, Birmingham, United Kingdom; 6 NIHR Birmingham Biomedical Research Centre, University Hospitals Birmingham NHS Foundation Trust and University of Birmingham, Birmingham, United Kingdom; 7 UCSF Benioff Children’s Hospital Oakland Research Institute, Oakland, California

## Abstract

Steroid biosynthesis and metabolism are reflected by the serum steroid metabolome and, in even more detail, by the 24-hour urine steroid metabolome, which can provide unique insights into alterations of steroid flow and output indicative of underlying conditions. Mass spectrometry–based steroid metabolome profiling has allowed for the identification of unique multisteroid signatures associated with disorders of steroid biosynthesis and metabolism that can be used for personalized approaches to diagnosis, differential diagnosis, and prognostic prediction. Additionally, steroid metabolome analysis has been used successfully as a discovery tool, for the identification of novel steroidogenic disorders and pathways as well as revealing insights into the pathophysiology of adrenal disease. Increased availability and technological advances in mass spectrometry–based methodologies have refocused attention on steroid metabolome profiling and facilitated the development of high-throughput steroid profiling methods soon to reach clinical practice. Furthermore, steroid metabolomics, the combination of mass spectrometry–based steroid analysis with machine learning–based approaches, has facilitated the development of powerful customized diagnostic approaches. In this review, we provide a comprehensive up-to-date overview of the utility of steroid metabolome analysis for the diagnosis and management of inborn disorders of steroidogenesis and autonomous adrenal steroid excess in the context of adrenal tumors.

**Essential Points**

- The steroid metabolome reflects biosynthesis, metabolism, and excretion of steroid hormones and readily reveals underlying conditions altering steroid flow and output
- The 24-hour urine steroid metabolome can be used to measure global steroid metabolism and net steroid output
- Unique urine steroid metabolome signatures (“steroid fingerprints”) result from inborn disorders of steroid biosynthesis and conditions associated with autonomous adrenal steroid excess and can be used for diagnostic purposes
- The combination of mass spectrometry–based steroid profiling with machine learning–based data analysis has created a powerful discovery tool, steroid metabolomics, which offers the prospect of personalized approaches to diagnosis, prognostic prediction, and therapy

The serum and urine steroid metabolomes provide significant insights into the biosynthesis, metabolism, and excretion of steroid hormones and readily reveal underlying enzymatic deficiencies associated with steroidogenesis. Although alterations in the steroid metabolome have been used to diagnose inborn errors in steroidogenesis for several decades, recent advances have refocused attention on the capabilities of steroid metabolome profiling. The combination of mass spectrometry–based steroid profiling with machine learning–based data analysis has created a powerful discovery tool, steroid metabolomics, highly suited as a diagnostic biomarker approach (1–4). Furthermore, novel technological developments have facilitated the development of high-throughput multisteroid profiling methods soon to reach clinical practice (5–7).

Adrenal cortex, gonads, and placenta are the primary sites of *de novo* steroidogenesis from cholesterol (Fig. 1). Some of the resulting steroids can directly bind and activate steroid receptors in target cells of steroid action, whereas others require downstream activation but may also be inactivated or diverted to other steroid pathways. This intracellular steroid prereceptor and postreceptor metabolism has also been termed “intracrinology” (8), explaining why circulating steroid concentrations are often not representative of observed biological hormone activity. Moreover, adrenal steroidogenesis exhibits a diurnal rhythm and, as a result, single–time point serum steroid measurements only provide snapshots. This problem is circumvented by analyzing 24-hour urine collections, facilitating quantitation of the net 24-hour steroid output.

**Figure 1.**
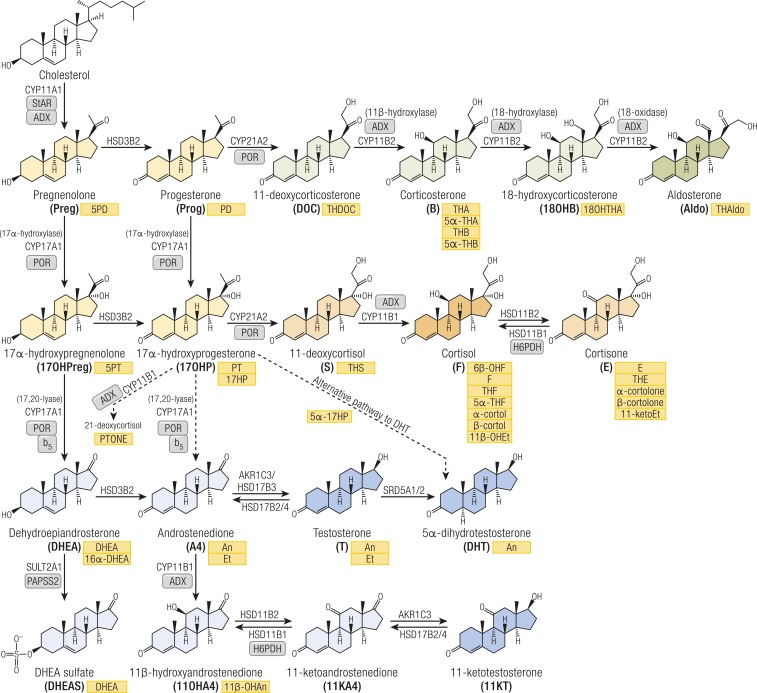
Schematic overview of steroidogenesis and corresponding urine steroid metabolites. Steroids are color-coded according to their bioactivity or commitment to a specific pathway: general precursors (yellow), mineralocorticoid precursors (light green), active mineralocorticoid (dark green), glucocorticoid precursors and inactive metabolite (light orange), active glucocorticoid (dark orange), androgen precursors (light blue), and active androgens (dark blue). Corresponding urinary metabolites are shown in yellow boxes. Arrows are labeled with the catalyzing enzyme and isoform. Essential cofactor proteins are also indicated: ADX, adrenodoxin; b_5_, cytochrome b_5_; PAPSS2, PAPS synthase 2; PRO, cytochrome P450 oxidoreductase; StAR, steroidogenic acute regulatory protein.

Both endogenous and exogenous steroids undergo hepatic metabolism (9), with phase 1 reactions altering the biological activity by adding or revealing functional groups that can function as targets for subsequent conjugation (phase 2) reactions. Ultimately, this results in steroid inactivation and increased water solubility, facilitating urinary excretion, which accounts for ∼80% of steroid excretion. Urine steroid metabolites originate from distinct circulating steroids (Fig. 1; Table 1); the 24-hour urine steroid metabolome serves as a magnifying glass, facilitating the detection of alterations in steroid biosynthesis or metabolism and, thus, of underlying disorders.

**Table 1.** Urine Steroid Metabolites as Assessed by Gas Chromatography–Mass Spectrometry (GC-MS)

No.	Abbreviation	Common Name	Chemical Name	Metabolite of
Androgen metabolites				
1	An	Androsterone	5*α*-Androstan-3*α*-ol-17-one	Androstenedione, testosterone, 5*α*-dihydrotestosterone
2	Et	Etiocholanolone	5*β*-Androstan-3*α*-ol-17-one	Androstenedione, testosterone
Androgen precursor metabolites				
3	11*β*-OHAn	11*β*-Hydroxyandrosterone	5*α*-Androstane-3*α*,11*β*-diol-17-one	11*β*-Hydroxyandrostenedione
4	DHEA	Dehydroepiandrosterone	5-Androsten-3*β*-ol-17-one	DHEA + DHEA sulfate (DHEAS)
5	16*α*-OHDHEA	16*α*-Hydroxy-DHEA	5-Androstene-3*β*,16*α*-diol-17-one	DHEA + DHEAS
6	5PT	Pregnenetriol	5-Pregnene-3*β*,17*α*,20*α*-triol	17*β*-Hydroxypregnenolone
7	5PD	Pregnenediol	5-Pregnene-3*β*,20*α*-diol	Pregnenolone
Mineralocorticoid and mineralocorticoid precursor metabolites				
8	THA	Tetrahydro-11-dehydrocorticosterone	5*β*-Pregnane-3*α*,21-diol-11,20-dione	11-Dehydrocorticosterone
9	5*α*-THA	5*α*-Tetrahydro-11-dehydrocorticosterone	5*α*-Pregnane-3*α*,21-diol-11,20-dione	11-Dehydrocorticosterone
10	THB	Tetrahydrocorticosterone	5*β*-Pregnane-3*α*,11*β*,21-triol-20-one	Corticosterone
11	5*α*-THB	5*α*-Tetrahydrocorticosterone	5*α*-Pregnane-3*α*,11*β*,21-triol-20-one	Corticosterone
12	THDOC	Tetrahydro-11-deoxycorticosterone	5*β*-Pregnane-3*α*,21-diol-20-one	11-Deoxycorticosterone
13	18OHTHA	18-Hydroxy-tetrahydro-11-dehydrocorticosterone	5*β*-Pregnane-3*α*,18,21-triol-11, 20-dione	18-Hydroxycorticosterone
14	THAldo	3*α,*5*β*-Tetrahydroaldosterone	5*β*-Pregnane-3*α*,11*β*,21-triol-20-one-18-al	Aldosterone
15	18OHF	18-Hydroxycortisol	4-Pregnene-11*β*,17*α*,18,21-tetrol-3,20-dione	Cortisol (hybrid steroid generated by CYP11B2 18-hydroxylation)
16	18oxoF	18-Oxo-cortisol	4-Pregnene-11*β*,17*α*,21-triol-3,20-dione-18-al	Cortisol (hybrid steroid generated by CYP11B2 18-oxidation)
17	18oxoTHF	18-Oxo-tetrahydrocortisol	5*β*-Pregnane-3*α*,11*β*,17*α*,21-tetrol-20-one-18-al	Cortisol (hybrid steroid tetrahydro metabolites)
Glucocorticoid precursor metabolites				
18	PD	Pregnanediol	5*β*-Pregnane-3*α*,20*α*-diol	Progesterone
19	5*α*-17HP	17*α*-Hydroxy-3*α*,5*α*-pregnanolone	5*α*-Pregnane-3*α*,17*α*-diol-20-one	17*α*-Hydroxyprogesterone
20	17HP	17*α*-Hydroxypregnanolone	5*β*-Pregnane-3*α*,17*α*-diol-20-one	17*α*-Hydroxyprogesterone
21	PT	Pregnanetriol	5*β*-Pregnane-3*α*,17*α*,20*α*-triol	17*α*-Hydroxyprogesterone
22	PTONE	Pregnanetriolone	5*β*-Pregnane-3*α*,17*α*,20*α*-triol-11-one	21-Deoxycortisol
23	THS	Tetrahydro-11-deoxycortisol	5*β*-Pregnane-3*α*,17*α*,21-triol-20-one	11-Deoxycortisol
Glucocorticoid metabolites				
24	F	Cortisol	4-Pregnene-11*β*,17*α*,21-triol-3,20-dione	Cortisol
25	6*β*-OHF	6*β*-Hydroxycortisol	4-Pregnene-6*β*,11*β*,17*α*,21-tetrol-3, 20-dione	Cortisol
26	THF	Tetrahydrocortisol	5*β*-Pregnane-3*α*,11*β*,17*α*,21-tetrol-20-one	Cortisol
27	5*α*-THF	5*α*-Tetrahydrocortisol	5*α*-Pregnane-3*α*,11*β*,17*α*,21-tetrol-20-one	Cortisol
28	*α*-Cortol	*α*-Cortol	5*β*-Pregnane-3*α*,11*β*,17*α*,20*α*,21-pentol	Cortisol
29	*β*-Cortol	*β*-Cortol	5*β*-Pregnane-3*α*,11*β*,17*α*,20*β*,21-pentol	Cortisol
30	11*β*-OHEt	11*β*-Hydroxyetiocholanolone	5*β*-androstane-3*α*,11*β*-diol-17-one	Cortisol
31	E	Cortisone	4-Pregnene-17*α*,21-diol-3,11,20-trione	Cortisone
32	THE	Tetrahydrocortisone	5*β*-Pregnene-3*α*,17*α*,21-triol-11,20-dione	Cortisone
33	*α*-Cortolone	*α*-Cortolone	5*β*-Pregnane-3*α*,17*α*,20*α*,21-tetrol-11-one	Cortisone
34	*β*-Cortolone	*β*-Cortolone	5*β*-Pregnane-3*α*,17*α*,20*β*,21-tetrol-11-one	Cortisone
35	11ketoEt	11-Ketoetiocholanolone	5*β*-Androstan-3*α*-ol-11,17-dione	Cortisone

Traditionally, gas chromatography–mass spectrometry (GC-MS) has been employed for comprehensive urine steroid metabolite profiling. Serum steroids are now increasingly analyzed by ultra-HPLC–tandem mass spectrometry (UHPLC-MS/MS), overtaking the use of immunoassays, which are increasingly recognized as compromised by cross-reactivity. In the routine clinical biochemistry context, UHPLC-MS/MS is primarily used for single steroid analysis; however, recent years have seen the emergence of multisteroid mass spectrometry analysis of serum and plasma steroids, and very recently, also of urine steroid metabolites. The translational application of steroid metabolomics has not only facilitated novel diagnostic biomarker approaches, but also facilitated the elucidation of novel steroid pathways and their roles in human disease, the discovery of steroidogenic disorders, as well as the more fine-grained categorization of autonomous steroid excess. In this review, we provide a comprehensive up-to-date overview of the distinct steroid metabolome signatures associated with disorders of steroid biosynthesis and metabolism, summarizing current knowledge about their utility for diagnosis, differential diagnosis, and prognostic prediction.

## Steroid Metabolome Signatures of Inborn Errors of Steroid Biosynthesis and Metabolism

### The steroid metabolome in congenital adrenal hyperplasia

The variants of congenital adrenal hyperplasia (CAH) comprise five autosomal recessive inborn disorders defined by glucocorticoid deficiency resulting from inactivating mutations in enzymes involved in adrenal steroidogenesis (10). Reduced cortisol feedback within the hypothalamic–pituitary–adrenal (HPA) axis drives continuous stimulation of the adrenal cortex by pituitary ACTH, with subsequent adrenocortical hyperplasia and enhanced activity of the unaffected adrenal steroidogenic pathways. Dependent on the position of the enzymatic block, mineralocorticoid and androgen production can be decreased, increased, or normal, respectively.

#### CAH due to CYP21A2 deficiency

**Figure 2.**
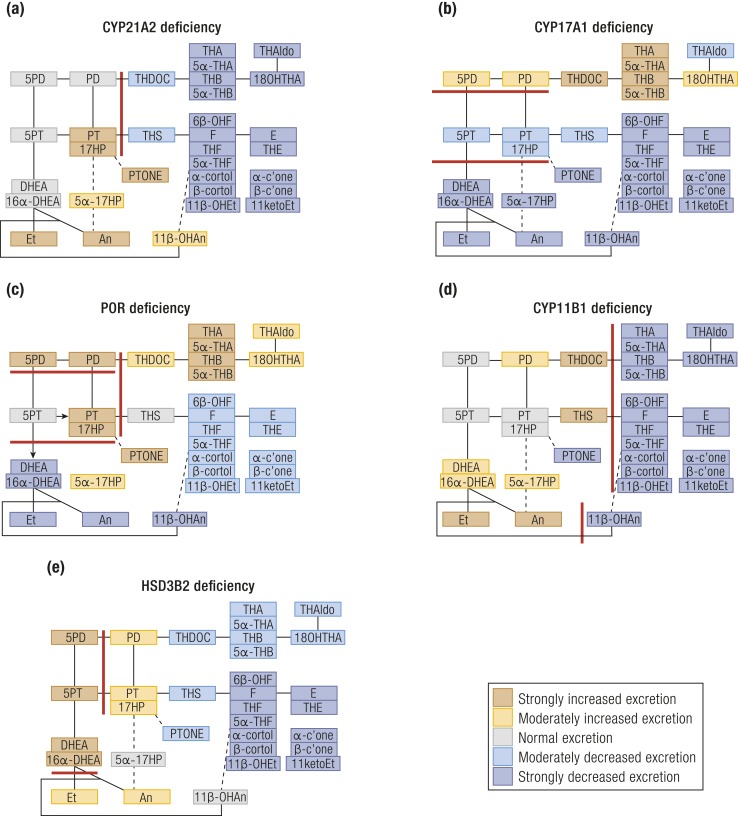
**(**a–e) Schematic visualization of urine steroid metabolome signatures in the five variants of CAH. (a) CYP21A2, (b) CYP17A1, (c) POR, (d) CYP11B1, and (e) HSD3B2 deficiencies. The figure depicts the changes in the major urine steroid metabolites relative to the reference range median of each metabolite and does not represent overall quantitative excretion. Steroid metabolites are mapped onto the steroidogenic pathways leading to mineralocorticoid, glucocorticoid, and androgen biosynthesis as shown in Fig. 1. Data derived from (13–28).

More than 90% of CAH cases are caused by mutant 21-hydroxylase (CYP21A2) (10), a key enzyme in glucocorticoid and mineralocorticoid biosynthesis (Fig. 1); the presence and severity of loss of cortisol and aldosterone production CYP21A2 deficiency is 17OHP, but 17OHPreg, Prog, and Preg are also increased (12). In the absence of CYP21A2 activity, CYP11B1 atypically converts 17OHP to 21-deoxycortisol. Therefore, diagnostic ratios of the urinary metabolites of 21-deoxycortisol (PTONE) or 17OHP (PT and 17HP) over glucocorticoid metabolites are invaluable for the diagnosis of CYP21A2 deficiency (13) [Fig. 2A (13–28); Table 2 (13, 26, 29, 30)].

**Table 2.** Substrate/Product Ratios of Urine Steroid Metabolites Used for the Biochemical Diagnosis of Inborn Errors of Steroidogenesis

Enzymatic Activity	Enzymes Involved	Ratio
21-Hydroxylase	CYP21A2 and POR	100*PTONE/(THE+THF+5*α*-THF)
		(17HP+PT)/(THE+THF+5*α*-THF)
17*α*-Hydroxylase	CYP17A1 and POR	(THA+5*α*-THA+THB+5*α*-THB)/
		(THE+THF+5*α*-THF)
		(THA+5*α*-THA+THB+5*α*THB)/(An+Et)
17,20-Lyase	CYP17A1 and CYB5A and POR	5PT/DHEA
		(17HP+PT)/(An+Et)
P450 oxidoreductase	POR	PD/(THE+THF+5*α*-THF)
		5PD/(THE+THF+5*α*-THF)
11*β*-Hydroxylase	CYP11B1	100*THS/(THE+THF+5*α*-THF)
3*β*-Hydroxysteroid dehydrogenase	HSD3B2	DHEA/(THE+THF+5*α*-THF)
		5PT/(THE+THF+5*α*-THF)
		5PT/PTONE
11*β*-Hydroxysteroid dehydrogenase type 2	HSD11B2	F/E
		(THF+5*α*-THF)/THE
		Cortols/cortolones
		(F+E)/(THF+5*α*-THF+THE)
11*β*-Hydroxysteroid dehydrogenase type 1	HSD11B1/H6PDH	THE/(THF+5*α*-THF)
		Cortolones/cortols
5*α*-Reductase type 2	SRD5A2	Et/An
		THB/5*α*-THB
		THF/5*α*-THF
17*β*-Hydroxysteroid dehydrogenase type 3	HSD17B3	(An+Et)/(THE+THF+5*α*-THF)
		An/Et

The prefix 100* indicates that steroid values are to be multiplied by 100 before calculating the respective steroid ratio. Ratios derived from (13, 26, 29, 30).

For all abbreviations, see Table 1.

In the past, diagnosis of CYP21A2 deficiency has been challenging in neonates and preterm infants, as 17OHP radioimmunoassay results are compromised by cross-reactivity of abundant neonatal 3*β*-OH-Δ^5^ steroids (31). When using GC-MS, polar 17OHP metabolites such as 5*β*-pregnane-3*β*,15*β*,17*α*-triol-20-one (13, 32, 33), as well as the ratio of PTONE over 6*α*-hydroxylated cortisone metabolites, can help discriminate affected from unaffected infants (13, 34), with additional diagnostic value provided by 5*α*-pregnane-3*β*,16*α*,17*α*-triol-7,20-dione and 5*β*-pregnane-3*α*,15*β*,17*α*-triol-20-one (35, 36).

**Figure 3.**
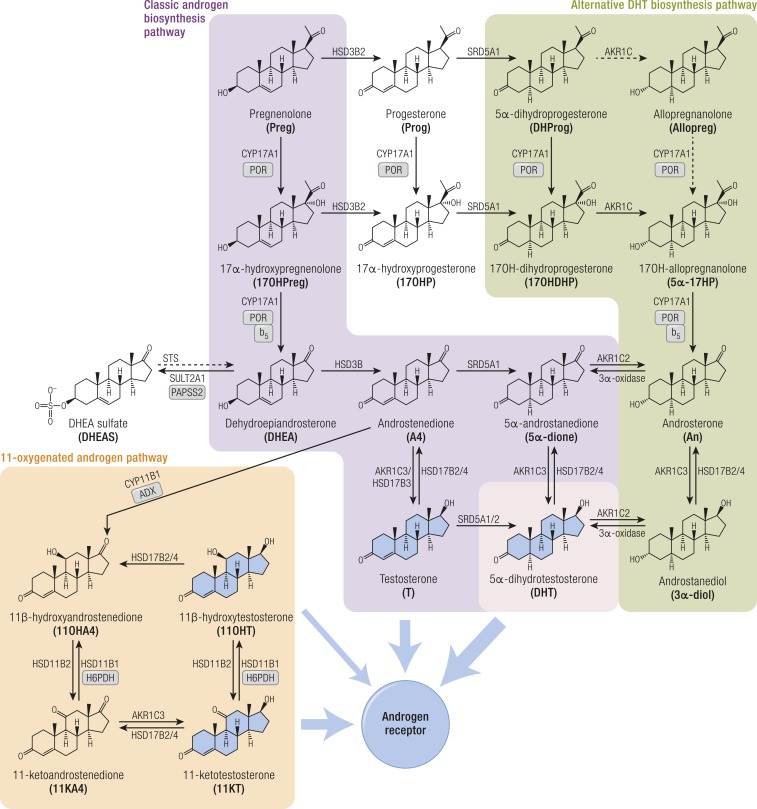
Schematic overview of the three major pathways of human androgen biosynthesis. The classic, alternative, and 11-oxygenated androgen pathways are each shown in different colors. Androgens that activate the androgen receptor are shown with broad blue arrows leading from them. Other arrows are labeled with the catalyzing enzyme and isoform where appropriate. Essential cofactor proteins are also indicated: b_5_, cytochrome b_5_; PAPSS2, PAPS synthase 2; POR, cytochrome P450 oxidoreductase.

Biologically active androgens are increased in CYP21A2 deficiency, driven by the accumulation of precursor steroids prior to the enzymatic block, feeding into all three major androgen biosynthesis pathways: the classic androgen pathway, the alternative pathway to DHT, and the 11-oxygenated androgen pathway (Fig. 3). The accumulation of 17OHP increases atypical conversion of 17OHP to androstenedione (A4) by CYP17A1 17,20-lyase activity, which physiologically has a much higher preference for the conversion of 17OHPreg to DHEA (29). Accumulating 17OHP also drives increased androgen production by the alternative DHT pathway (26), and increased A4 feeds enhanced 11-oxygenated androgen pathway activity (24) (Fig. 3). An and Et are typically raised in urines of untreated or poorly controlled patients, although note that Et is derived solely from the classic pathway, whereas An can be derived from both classic and alternative androgen pathways (Table 1); urinary DHEA(S) excretion is usually normal or only mildly elevated. Alternative DHT pathway activity is reflected by 5*α*17HP and An (22, 26), whereas increased 11-oxygenated pathway activity is reflected by the major metabolite of 11OHA4, 11*β*-OHAn (22, 25, 26) (Figs. 2A and 3; Table 1).

#### CAH due to CYP17A1 deficiency

CYP17A1 is vital for both glucocorticoid and androgen biosynthesis via its 17*α*-hydroxylase and 17,20-lyase activities, respectively. Therefore, CYP17A1 deficiency mostly presents with combined glucocorticoid and sex steroid deficiency; HPA axis upregulation drives increased mineralocorticoid production via the only remaining functional adrenocortical biosynthesis pathway. Glucocorticoid deficiency is rarely life-threatening in CYP17A1 deficiency, as increased corticosterone exerts some glucocorticoid receptor activation (11, 12).

Affected patients more commonly present with symptoms of sex steroid deficiency with associated hypertension due to very high levels of DOC. Because not only female but also chromosomally male patients appear phenotypically female, it is not uncommon that diagnosis is not made until puberty. In CYP17A1 deficiency, serum DHEA and A4 are low at baseline and after adrenal cosyntropin stimulation (11). Urine steroid profiling (Fig. 2B) shows a substantial reduction of all androgen metabolites, and diagnostic ratios (Table 2) indicate largely abolished 17,20-lyase activity (15, 18).

Although milder forms of CYP17A1 deficiency have been reported, where only sex steroid production seems to be present (so-called “isolated 17,20-lyase deficiency”), stimulated cortisol levels do not rise sufficiently in these patients, indicating glucocorticoid deficiency (15, 37). Similarly, urine steroid profiling in these milder cases suggests attenuation of 17*α*-hydroxylase activity, assessed by the ratio of mineralocorticoid over glucocorticoid metabolites (15, 19), which is the most important diagnostic ratio for this disorder (Table 2). In classic forms with severe CYP17A1 deficiency, DOC and corticosterone metabolites are markedly increased, with mildly increased Preg and Prog metabolites (38). Circulating 17OHP is significantly decreased, whereas cortisol and aldosterone levels remain normal (17, 39). In neonates with CYP17A1 deficiency, the 11-keto corticosterone metabolite 11-dehydrocorticosterone is dominant in newborns and, therefore, urine steroid metabolites increased in CYP17A1 deficiency include THA, 5*α*-THA, and 6*α*-hydroxy-11-dehydro-tetrahydrocorticosterone (6*α*-OHTHA), with the latter the most important quantitative marker. Thus, useful diagnostic urine steroid ratios in neonates include 6*α*-OHTHA/cortisol metabolites and 16*α*-hydroxypregnenolone/16*α*-OHDHEA (13).

#### CAH due to P450 oxidoreductase deficiency

P450 oxidoreductase (POR) is the crucial electron donor for microsomal cytochrome P450 (CYP) enzymes, including the steroidogenic enzymes CYP21A2, CYP17A1, and, to a lesser degree, CYP19A1 (11, 12). The discovery of the molecular basis of POR deficiency (PORD) in 2004 (40, 41) solved the puzzle previously posed by patients with a unique urine steroid profile published 20 years earlier, indicating the concurrent presence of CYP21A2 and CYP17A1 deficiencies (42). Most patients have normal baseline cortisol but respond insufficiently to cosyntropin (16), indicative of partial glucocorticoid deficiency, which requires stress dose cover with glucocorticoids in case of intercurrent illness, major stress, or surgery. Mineralocorticoid production is preserved or enhanced, with hypertension typically manifesting in adulthood (16). Preg and Prog are characteristically increased, whereas 17OHP is only mildly elevated. The impairment of other enzymes involved in cholesterol biosynthesis (CYP51A1, SQLE) (30, 43) and retinoic acid metabolism (CYP26A1, CYP26B1, CYP26C1) (44, 45) results in skeletal and multiple other malformations resembling the Antley-Bixler phenotype (16). PORD also results in decreased hepatic drug metabolism, due to reduced capacity of CYP3A4, but also CYP1A2, CYP2D6, CYP2C9, and CYP2C19 (46).

The urine steroid metabolome in PORD shows characteristically increased Preg and Prog metabolites (PD and 5PD; Fig. 2C, Table 1), which together with the increased metabolites attributed to partial CYP21A2 and CYP17A1 deficiencies establishes the diagnosis (14, 16, 23, 27) (Table 2). 5PD, excreted as a bis-sulfate, is particularly prominent in neonates and young infants with PORD (47).

In PORD, DSD has been reported in individuals of both chromosomal sexes, and patients can present as virilized females (46,XX DSD) as well as undermasculinized males (46,XY DSD). This paradox has been explained by the alternative pathway to DHT (Fig. 3), proposed to be mostly active during fetal life, with very low or absent activity in the postnatal situation (40). In individuals with 46,XX, androgen excess generated via the alternative pathway may cause virilization; equally, androgen biosynthesis may be insufficient to masculinize external genitalia in individuals with 46,XY, depending on the effect of the underlying mutations (16, 48, 49). *POR* mutations allowing for significant residual alternative pathway activity present with 46,XY DSD and normal male genitalia in individuals with 46,XY, whereas major loss-of-function *POR* mutations result in normal female phenotype and 46,XY DSD. Maternal virilization is also a characteristic feature in many but not all pregnancies with affected babies (41).

Prenatal diagnosis at midpregnancy is straightforward (50, 51). Attenuated activity of POR results in multiple enzyme deficiencies en route to estriol. As a result, unconjugated serum estriol is typically very low. Decreased CYP17A1 activity yields excess excretion of 5*α*-pregnane-3*β*,20*α*-diol (3*β*5*α*-PD) bis-sulfate, a fetal pregnenolone metabolite, increasing the urinary 3*β*5*α*-PD/estriol ratio (50). Upregulation of the alternative pathway upregulates 5*α*-17HP and An production, and consequently increased urinary An/Et and 5*α*-17HP/17HP ratios (50). LC-MS/MS analysis of conjugated urine steroids recently revealed 3*β*5*α*-PD bis-sulfate and estriol glucuronides to be useful in the prenatal diagnosis of this disorder (47).

#### CAH due to CYP11B1 deficiency

CYP11B1 catalyzes key reactions in the mineralocorticoid and glucocorticoid pathways. CYP11B1 deficiency results in cortisol deficiency, mineralocorticoid excess, and androgen excess. The marker steroid is 11-deoxycortisol, accumulating prior to the enzymatic block. DOC also accumulates due to continuous ACTH stimulation, resulting in arterial hypertension (11, 12), with CYP11B2-mediated conversion of DOC to corticosterone not sufficient to compensate for loss of CYP11B1 function.

Serum A4 and testosterone (T) are increased in untreated or poorly controlled individuals, whereas 11-oxygenated androgens are characteristically absent, with low excretion of the 11OHA4 metabolite 11*β*-OHAn (20, 52). The urine steroid metabolome is dominated by THS; diagnosis is facilitated by the ratio of THS over glucocorticoid metabolites (Table 2; Fig. 2D). THDOC is also increased. In contrast to CYP21A2 deficiency, PTONE is low in CYP11B1 deficiency, as 17OHP cannot be 11*β*-hydroxylated. In neonates, high 6*α*-hydroxylase activity increases 6*α*-hydroxytetrahydrotetrahydro-11-deoxycortisol (6*α*-OHTHS) excretion, which adds diagnostic value (13, 53).

#### CAH due to HSD3B2 deficiency

HSD3B2 *(*Δ^5-4^ isomerase) is crucial to the production of all three major adrenal steroid classes as well as gonadal androgens (Fig. 1). Deficiency of HSD3B2, therefore, leads to reduced mineralocorticoid, glucocorticoid, and sex steroid production. HPA axis activation drives precursor accumulation, in particular 17OHPreg (54). Classically, patients manifest early in the neonatal period with severe salt-wasting adrenal insufficiency, but broad phenotypic variation is reported (54). The HSD3B1 isoform, expressed in the placenta several peripheral tissues, converts accumulating ∆^5^ steroids such as 17OHPreg or DHEA to 17OHP and active androgens, respectively. Therefore, serum 17OHP can be elevated and individuals with 46,XX can present virilized. In urine, the 17OHPreg metabolite 5PT and DHEA are raised, and downstream metabolites of all steroidogenic pathways are reduced (21) (Fig. 2E). Surprisingly, excretion rates of PT, 17HP, and PD are elevated, similar to CYP21A2 deficiency; however, in CYP21A2 deficiency, the 5PT/PTONE ratio is typically low (<1.5), whereas it is high (>35) in HSD3B2 deficiency (14, 23) (Table 2). In neonates, elevated 5PT is also an important marker (13), with added diagnostic value of 5-pregnene-3*β*,15*β*,17*α*-triol-20-one and its 20-reduced metabolite (13, 55).

### Inborn disorders of androgen biosynthesis and metabolism

#### SRD5A2 deficiency

SRD5A2 catalyzes the final activating step of the classic androgen biosynthesis pathway, the 5*α*-reduction of T to the more potent DHT (Fig. 1). In chromosomally male individuals, SRD5A2 deficiency presents with undermasculinization, that is, ambiguous genitalia at birth (46,XY DSD) (56). SRD5A2 deficiency has elucidated the crucial role of DHT in male genital skin, as in its absence, external genital masculinization does not occur. If not diagnosed and appropriately treated, affected individuals are inadvertently raised as females and, owing to increased expression of SRD5A1 in the genital area at pubertal age, may then present with significant virilization and phallus growth (57, 58).

Serum DHT is low/undetectable at baseline and after human chorionic gonadotropin (hCG) stimulation, with inappropriately high T. However, establishing the diagnosis from the hCG-stimulated serum T/DHT ratio is highly challenging, due to the very low circulating DHT concentrations, in particular in infants and prepubertal children (58–60).

**Figure 4.**
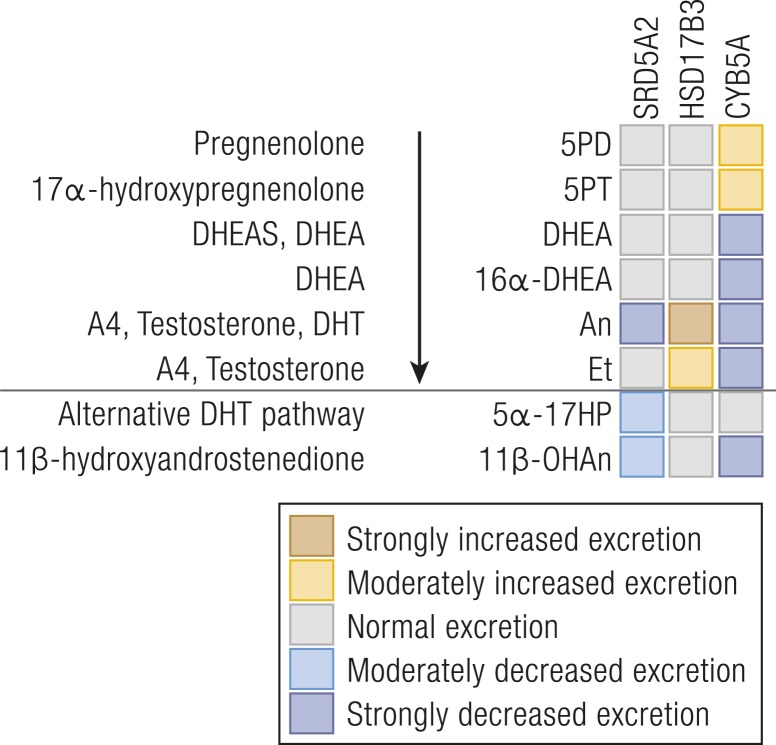
Heat map visualization of urine steroid metabolome signatures associated with inborn disorders of androgen biosynthesis. The figure depicts the changes in the major urine steroid metabolites associated with androgen biosynthesis relative to the reference range median of each steroid metabolite and does not represent overall quantitative excretion. For explanation of the link between precursors, active steroids, and their metabolites, please see Fig. 1. Data derived from (15, 61, 62).

The diagnosis from urine steroid profiling is straightforward [Fig. 4 (15, 61, 62)], with most robust information provided by diagnostic ratios of 5*α*-reduced over 5*β*-reduced glucocorticoid and mineralocorticoid metabolites (Table 2) (61), owing to the abundance of those metabolites compared with androgen metabolites, in particular in prepubertal children. Diagnosis can be difficult in neonates and may not be reliably established from urine steroid profiling until the age of 3 months, owing to high activity of the SRD5A1 isoform and lower excretion rates of cortisol metabolites during this stage of development (23, 63, 64).

#### HSD17B3 deficiency

Inactivating HSD17B3 mutations results in failure to convert A4 to T in the fetal testis, causing male undermasculinization (46,XY DSD) (65). Owing to increasing expression of other HSD17B isoforms capable of using A4 as a substrate, affected individuals tend to experience significant virilization at the time of puberty.

Serum T is low and A4 increased, both at baseline and after hCG stimulation. An hCG-stimulated T/A4 ratio <0.8 has been suggested as diagnostic in children (66). However, false-positive results have been reported in other disorders of T biosynthesis or structural abnormalities of the testis, that is, Leydig cell hypoplasia (67, 68).

There is a paucity of published data on urine steroid profiling in HSD17B3 deficiency. One might expect that measuring a pair of 17-keto and 17-hydroxy ratios would allow for diagnosis, but in our experience this is not the case, possibly explained by the activities of other HSD17B isoforms. Affected children excrete high levels of the major androgen metabolites, particularly An, increasing the An/Et ratio (Fig. 4). Otherwise, the steroid metabolome is largely normal (14, 23, 62). A notable feature, useful diagnostically, is an increased androgen-over-glucocorticoid metabolite ratio (An+Et/THE+THF+5aTHF) (Table 2). Age-appropriate controls are important when utilizing this ratio.

#### CYB5A deficiency (true isolated 17,20-lyase deficiency)

Cytochrome b_5_ (CYB5A) is a modulator crucially required for CYP17A1 17,20-lyase activity (Fig. 1) and hence essential for sex steroid production. Individuals with 46,XY with inactivating CYB5A mutations present with 46,XY DSD at birth, and girls with 46,XX present with lack of adrenarche, pubertal development, and primary gonadal failure in adolescence. Mild but clinically asymptomatic methemoglobinemia is also observed due to the role of CYB5A in Hb metabolism (69).

Serum sex steroids DHEA(S), A4, and T as well as 17*β*-estradiol are undetectable at baseline and after cosyntropin or hCG stimulation (15, 70). Urine steroid profiling data are available in three siblings (15): excretion rates of the 17OHPreg metabolite 5PT are increased, with reduced androgen excretion but normal glucocorticoids and mineralocorticoids (Fig. 4). In contrast to 17,20-lyase deficiency in the context of CYP17A1 deficiency (see “CAH due to CYP21A2 deficiency” above), which always comes with a degree of impairment of CYP17A1 17*α*-hydroxylase activity, CYB5A deficiency represents “true” isolated 17,20-lyase deficiency (15, 70); increased steroid ratios for 17,20-lyase activity with normal ratios for 17*α*-hydroxylase activity are diagnostic (Table 2).

### Inborn disorders of sulfation and desulfation

#### Steroid sulfatase deficiency

Steroid sulfatase (STS) cleaves the sulfate moiety off a variety of sterol and steroid sulfates, including DHEAS (71). Patients with STS deficiency (STSD) are mainly affected by X-linked ichthyosis characterized by dark-brown scaling of the skin due to accumulating cholesterol sulfate in the epidermis (72, 73). STSD could theoretically contribute to a reduced desulfation of DHEAS and, therefore, reduced availability of DHEA for downstream activation to T and DHT. However, several previous studies did not find clinically significant androgen deficiency in affected patients (74–78). A study exploring the serum and urine steroid metabolome in prepubertal and postpubertal children found decreased serum DHEA and T compared with matched controls (77). The urinary excretion of active androgen metabolites An and Et, however, was similar in STSD and controls, possibly due to an upregulation of systemic 5*α*-reductase activity, as indicated by an increased urinary 5*α*-THF/THF ratio (77). The serum DHEA/DHEAS ratio reflective of global STS activity was high in prepubertal controls and decreased during the course of puberty; in contrast, STSD patients showed low DHEA/DHEAS ratios both prepubertally and postpubertally (77). This suggests a physiological role of STS prior to puberty, possibly for fine tuning of tissue-specific androgen activation, no longer needed postpubertally in the presence of high gonadal androgen production.

Serum cholesterol sulfate is highly elevated in STS deficiency (79), and diagnosis can readily be achieved by UHPLC-MS/MS analysis of the intact conjugate (80, 81).

There are antenatal indicators of STSD, and prenatal diagnosis of an affected fetus is straightforward. It is the most common cause of low estriol production during pregnancy, with free urine estriol values typically very low. The placenta of an affected fetus is unable to desulfate the fetal estriol precursors (*e.g.*, 16*α*-OHDHEAS), thus preventing unconjugated 16*α*-OHDHEA formation and its conversion to 16*α*-hydroxyandrostenedione. Concomitantly, increased maternal excretion of all Δ^5^ steroid sulfates, particularly the two precursors of estriol, 16*α*-OHDHEAS and androstenetriol sulfate (5-androstene-3*β*,16*α*,17*β*-triol sulfate), are characteristic of this disorder during pregnancy (82). The ratio of urinary 16*α*-OHDHEA/estriol measured by GC-MS is used for diagnosis (83). Moreover, UHPLC-MS/MS of intact steroid conjugates has recently been evaluated for prenatal STSD diagnosis (47). 16*α*-OHDHEA sulfate, 5-pregnene-3*β*,20*α*-diol bis-sulfate, 21-hydroxypregnenolone bis-sulfate, and estriol glucuronides were found to be effective diagnostic markers.

#### PAPSS2 deficiency (apparent DHEA sulfotransferase deficiency)

PAPSS2 generates the universal sulfate donor PAPS required by DHEA sulfotransferase (SULT2A1), which catalyzes sulfation of DHEA to DHEAS (Fig. 1) (84). PAPSS2 deficiency is a rare monogenic form of androgen excess caused by impaired DHEA sulfation, resulting in an increased downstream activation of unconjugated DHEA to androgens (85).

The seminal case of PAPSS2 deficiency was a girl with early-onset androgen excess who clinically presented with premature adrenarche at the age of 6 years, thereafter progressing to a polycystic ovary syndrome (PCOS)–like phenotype in adolescence (85). The key abnormality was low/undetectable serum DHEAS, but high levels of A4, T, and DHT. Low serum DHEAS is a common finding in patients with PAPSS2 deficiency (85–88).

Detailed investigations of steroid metabolism are available from two brothers with compound heterozygous *PAPSS2* mutations and their heterozygous parents (89). After an oral DHEA challenge, the brothers and their mother exhibited a subnormal rate of DHEAS generation, whereas DHEA generation and urinary androgen excretion increased, with evidence of increased 5*α*-reductase activity (Table 2) in the affected brothers and their mother (89). Of note, this mother and the mother of the first reported case were carriers of major loss-of-function *PAPSS2* mutations and presented clinically with PCOS.

### Inborn disorders of cortisol activation and inactivation

#### Cortisone reductase (HSD11B1) deficiency and apparent cortisone reductase (H6PDH) deficiency

Cortisone reductase deficiency (CRD) and apparent CRD (ACRD) are characterized by the inability to generate active cortisol from cortisone (Fig. 1), with the consequently activated HPA axis driving ACTH-mediated excess adrenal androgen secretion. CRD is caused by HSD11B1 deficiency (90) whereas ACRD results form a deficiency in the activity of H6PDH, which is essential for maintaining the reductive activity of HSD11B1 *in vivo* by the reduced form of NAD phosphate provision to the enzyme (91, 92).

**Figure 5.**
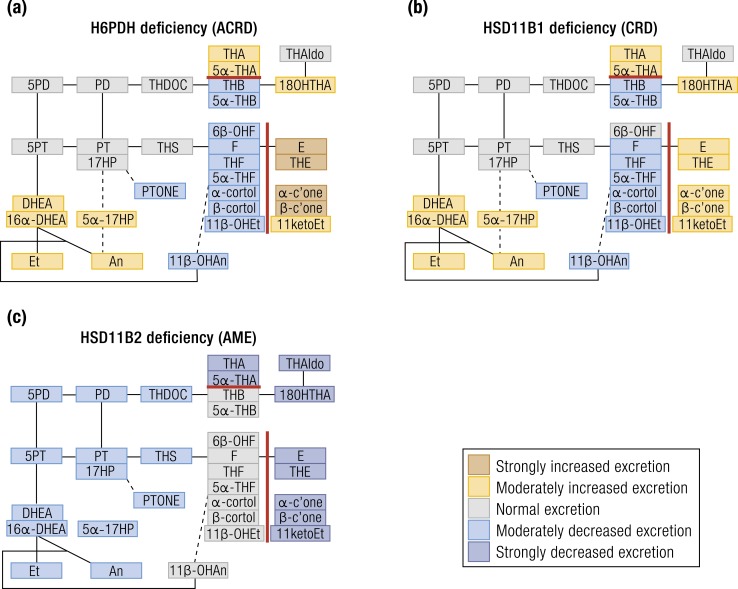
Heat map visualization of urine steroid metabolome signatures associated with HSD11B and H6PDH deficiencies. (a) H6PDH deficiency, (b) HSD11B1 deficiency, and (c) HSD11B2 deficiency. The figure depicts the changes in the major urine steroid metabolites relative to the reference range median of each metabolite and does not represent overall quantitative excretion. Steroid metabolites are mapped onto the steroidogenic pathways leading to mineralocorticoid, glucocorticoid, and androgen biosynthesis as shown in Fig. 1. Data derived from (90, 92, 95–100).

Clinically, CRD and ACRD present with a similar phenotype, with affected individuals developing androgen excess in childhood, coming to clinical attention with premature adrenarche in children of both sexes or in adolescent and young adult women with a PCOS-like phenotype (90, 92–94). Urine steroid profiling reveals distinct alterations in glucocorticoid metabolism accompanied by an overall increase of androgen excretion rates (90, 92, 95). Notably, the urinary excretion of cortisone metabolites is increased whereas cortisol metabolites are decreased (92) [Fig. 5A and 5B (90, 92, 95–100)], resulting in reduced ratios of cortisol over cortisone metabolites (Table 2).

Detailed analysis of CRD and ACRD cases suggest that the (5*α*-THF+THF)/THE ratio and the cortols/cortolones ratio may be used to distinguish the two conditions (≤0.1 for ACRD but 0.1 to 0.5 in CRD) (95). Total androgen metabolite excretion is increased; however, a distinction between CRD and ACRD based on androgen metabolites is not possible, although there is a tendency toward higher androgen output in CRD (95). Global 5*α*-reductase activity based on 5*α*-reduced over 5*β*-reduced THF is increased in most ACRD cases, but normal in CRD (95).

*“…machine learning–driven analysis of the urinary steroid excretion data allowed for rapid detection of a ‘malignant steroid fingerprint’….”*

#### HSD11B2 deficiency (apparent mineralocorticoid excess)

The central role of HSD11B2 is to protect mineralocorticoid target tissues from cortisol-mediated activation of the mineralocorticoid receptor (MR) (101). Therefore, HSD11B2 deficiency caused by inborn mutations or excess consumption of HSD11B2 inhibitors (carbenoxolone, licorice) (102, 103) leads to unwanted excess MR activation by cortisol, resulting in apparent mineralocorticoid excess (AME) (104). Clinically, HSD11B2 deficiency manifests with hypertension, hypokalemia, and low renin levels, but no evidence of excess mineralocorticoid secretion, with subnormal levels of aldosterone (99). Most patients are children presenting within the first year of life with failure to thrive, short stature, and hypokalemia leading to thirst, polydipsia, and polyuria (105).

Circulating cortisol remains normal due to increased negative HPA axis feedback. Circulating cortisone is decreased, which increases the cortisol/cortisone ratio (106), the key marker of HSD11B2 function (107). The urine steroid metabolome shows increased excretion of 11*β*-hydroxysteroids over the respective 11-ketosteroids (Fig. 5C).

The ratio of cortols/cortolones is increased (96, 97, 108), whereas 11ketoEt excretion is undetectable (98). The excretion of metabolites of DOC and aldosterone are subnormal (96) as, in fact, is the excretion of all steroids. Quantitatively, even the diagnostic “hyperproduced” 11*β*-hydroxy cortisol metabolites are at the lower end of the normal range for age.

Features of impaired cortisol clearance other than decreased cortisol 11-oxidation have also been observed in AME. An increased urinary 5*α*-THF/THF ratio indicates reduced AKR1D1 activity or a shunt of cortisol into the pathway of 5*α*-reduction of glucocorticoids (98, 99). There is also an impaired conversion of tetrahydro cortisol metabolites to their corresponding hexahydro metabolites, indicating defective reductive metabolism of the cortisol side chain. There is an increased excretion of urinary free cortisol and cortisol metabolites with an unreduced or incompletely reduced A-ring—for example, 6*β*-OHF and 5*α*-dihydrocortisol (96, 109). The urinary free cortisol–to–urinary free cortisone ratio has emerged as a more sensitive marker for AME than the ratio of (THF+5*α*-THF)/THE (100), as A-ring reduction to tetrahydro metabolites takes place mainly in the liver and their ratio may not accurately reflect renal HSD11B2 activity.

### Inborn mineralocorticoid deficiency and excess

#### CYP11B2 deficiency

CYP11B2 catalyzes the three final steps of aldosterone production, exerting sequential 11*β*-hydroxylase, 18-hydroxylase, and 18-oxidase activities (Fig. 1). *CYP11B2* mutations invariably result in loss of 18-oxidase activity, whereas the 18-hydroxylase activity can be either preserved or lost. In both cases, patients present with identical clinical features of mineralocorticoid deficiency: signs of hyponatremia, hyperkalemia, and hypovolemia, which can lead to shock and death. Plasma renin activity is increased in affected children, but it can be normal in adults (110, 111). As the two-step conversion of corticosterone to aldosterone was initially considered to be catalyzed by two different enzymes—corticosterone methyloxidase I (CMO I, 18-hydroxylase) and corticosterone methyloxidase II (CMO II, 18-oxidase)—a biochemical categorization of CYP11B2 deficiencies as CMO I and CMO II is still widely accepted.

##### 18-Hydroxylase deficiency (CMO I due to CYP11B2 deficiency).

**Figure 6.**
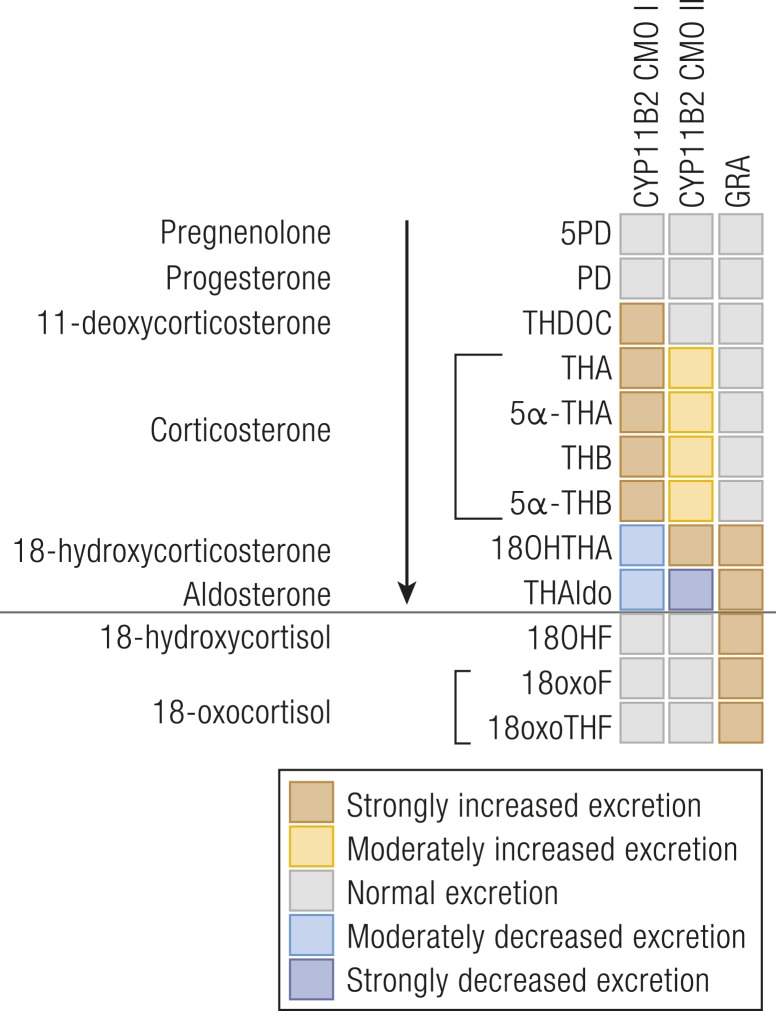
Heat map visualization of urine steroid metabolome signatures associated with inborn mineralocorticoid excess. The figure depicts the changes in the major urine steroid metabolites associated with mineralocorticoid biosynthesis relative to the reference range median of each metabolite and does not represent overall quantitative excretion. For explanation of the link between precursors, active steroids, and their metabolites, please see Fig. 1. Data derived from (50, 112–124).

18-hydroxylase deficiency results in low serum 18-hydroxycorticosterone (18OHB) and low to undetectable aldosterone, with concomitant accumulation of corticosterone. As a consequence, urinary excretion of 18OHTHA and THAldo is low whereas corticosterone metabolites are high [Fig. 6 (50, 112–124)].

THDOC may also be increased, whereas cortisol metabolite excretion is normal (112, 117). In neonates, 5*α*-THA is higher than 5*α*-THB, due to the dominance of the 11-keto derivatives, and 6*α*-OHTHA is also a quantitatively relevant metabolite, due to high neonatal 6*α*-hydroxylase activity (117).

##### 18-Oxidase deficiency (CMO II due to CYP11B2 deficiency).

In 18-oxidase deficiency, aldosterone levels are low whereas 18OHB levels are increased, in contrast to low 18OHB in CMO I. Serum corticosterone is normal to high, depending on the severity of 18-oxidase deficiency. Consequently, the urine metabolome shows low to undetectable THAldo, whereas 18OHTHA is significantly increased (Fig. 6). Corticosterone metabolite excretion is normal or increased, whereas cortisol metabolites are normal (118, 121, 122). The urinary ratio of THAldo/18OHTHA or plasma aldosterone/18OHB discriminate CMO I and CMO II conditions (119, 125, 126). When aldosterone is undetectably low and these ratios are incalculable, which can occur in CMO I conditions (127), the circulating 18OHB/B ratio or urinary 18OHTHA/THBs ratio can be used (123).

#### Pseudohypoaldosteronism

Pseudohypoaldosteronism (PHA) is a rare syndrome of systemic or renal mineralocorticoid resistance characterized by excessive aldosterone secretion, but clinical signs of hypoaldosteronism, which may result from genetic disorders, transient, or secondary salt-losing states (128, 129). PHA is characterized by increased plasma renin and aldosterone as well as increased urinary excretion of aldosterone metabolites (120, 130, 131). Unlike CYP11B2 deficiency CMO II, both 18OHTHA and THAldo are increased in neonates, and thus their ratio is unaffected in PHA, allowing the two conditions to be distinguished (Fig. 6) (119).

#### Glucocorticoid-remediable aldosteronism

Rare unequal crossover events between the *CYP11B1* and *CYP11B2* genes yield a hybrid *CYP11B* gene composed of the ACTH-responsive promoter and first exons of *CYP11B1* fused in-frame to the major part of *CYP11B2*, resulting in ACTH-driven mineralocorticoid production (124, 132). Mineralocorticoid excess is controlled by exogenous glucocorticoid administration, which suppresses endogenous ACTH, reflected in the name of the condition, glucocorticoid-remediable aldosteronism (GRA), sometimes also called familial hyperaldosteronism (FHA) type 1.

**Figure 7.**
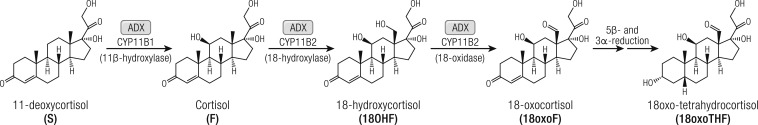
Schematic overview of the biosynthesis and downstream metabolism of the “hybrid steroids” 18-hydroxycortisol (18OHF) and 18-oxocortisol (18oxoF). Whereas 18OHF is excreted unmodified in urine, 18oxoF is primarily detected as its tetrahydro metabolite (18oxoTHF).

GRA is characterized by aldosterone excess with suppressed renin and increased production of 18-oxygenated cortisol “hybrid” metabolites, 18-hydroxycortisol (18OHF) and 18-oxocortisol (18oxoF) (Fig. 7) (133–135). Urinary excretion of aldosterone metabolites is increased (Fig. 6) (115, 124), with the urinary (18OHF+18oxoF)/THAldo ratio further supporting diagnosis (116); the urinary metabolite 18oxoTHF (Fig. 7) is characteristically increased.

## The Steroid Metabolome in Autonomous Adrenal Steroid Excess

### Autonomous adrenal cortisol excess

Clinically overt Cushing syndrome presents with characteristic signs and symptoms (facial plethora, broad and purplish stretch marks, easy bruising, proximal muscle weakness) but also less specific ones (centripetal obesity, acne, hirsutism, oligomenorrhea, edema, hypertension, type 2 diabetes, osteoporosis) (136, 137). Most cases of Cushing syndrome are due to excess stimulation of the adrenals by ACTH, either due to a pituitary tumor (= Cushing disease; 70% to 75%) or ectopic ACTH secretion (10% to 15%). However, in the remaining 10% to 15%, primary adrenal cortisol excess is the cause of disease, mostly due to a glucocorticoid-secreting adrenocortical adenoma (ACA) or, less frequently, an adrenocortical carcinoma (ACC) (136). More rarely, autonomous adrenal cortisol hypersecretion is caused by primary bilateral macronodular adrenal hyperplasia, due to *AMRC5* mutations (138), or primary pigmented nodular adrenocortical disease due to inactivating *PRKAR1A* mutations, affecting the regulatory subunit of the cAMP-dependent protein kinase A (136). Mutations in the *PRKACA* gene, encoding the catalytic subunit of protein kinase A, have been identified as a frequent cause of cortisol excess in unilateral adrenal adenomas due to somatic driver mutations, but also as a rare germline mutation underlying bilateral macronodular adrenal hyperplasia (139).

Adrenal masses are found incidentally in a large number of individuals, and it is estimated that 5% of the general population harbor an adrenal mass. Only 5% of those are cortisol-producing adenomas that manifest as adrenal Cushing syndrome. However, much larger numbers of ACAs are associated with mild autonomous cortisol excess (MACE), also previously termed subclinical Cushing syndrome (140–143). MACE presents with nonspecific signs and symptoms potentially related to cortisol excess, lacking the characteristic clinical Cushing features. The exact prevalence and clinical implications of this condition remain incompletely determined, but an association with metabolic comorbidities (obesity, type 2 diabetes, hypertension, osteoporosis) has been reported by retrospective studies (144–146) and a recent systematic review and meta-analysis (147).

**Figure 8.**
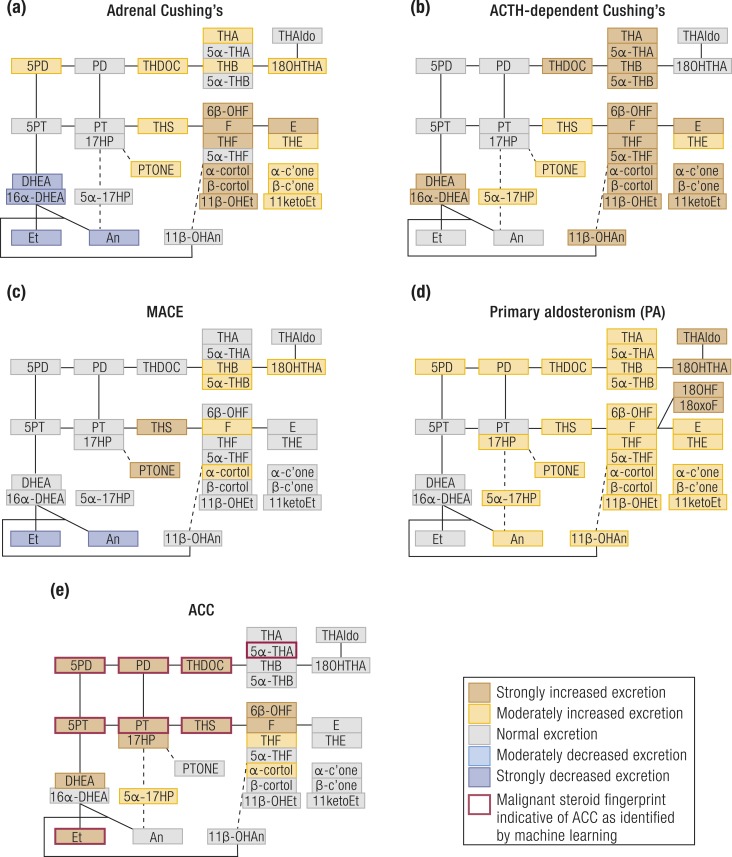
Schematic visualization of urinary steroid metabolome signatures associated with disorders causing autonomous adrenal steroid excess. (a) Adrenal Cushing syndrome, (b) ACTH-dependent Cushing syndrome, (c) MACE, (d) primary aldosteronism, and (e) ACC. The figure depicts the changes in the major urinary metabolites relative to the normal median of each steroid metabolite and does not represent overall quantitative excretion. Steroid metabolites are mapped onto the steroidogenic pathways leading to mineralocorticoid, glucocorticoid, and androgen biosynthesis as shown in Fig. 1. Data derived from (1, 148–155).

The urine steroid metabolome of patients with overt Cushing syndrome is characterized by excessive excretion of glucocorticoid and mineralocorticoid precursor metabolites, although androgen metabolites tend to be suppressed (148, 149) in adrenal Cushing but increased in ACTH-dependent Cushing syndrome (150, 151) [Fig. 8A and 8B (1, 148–155)]. Similar, albeit less pronounced changes are observed in patients with MACE (148) (Fig. 8C).

In Cushing syndrome, excess cortisol overwhelms the capacity of HSD11B2 (151, 152), the enzyme that inactivates cortisol to cortisone, resulting in a high ratio of urinary (THF+5*α*-THF)/THE (151). 5*α*-Reductase activity is mostly decreased in Cushing syndrome, as reflected by an increased THF/5*α*-THF ratio (151). The possibility that steroid profiling may be used diagnostically for the differential diagnosis of patients with Cushing syndrome has only been tentatively explored (150), and it is not known yet whether different genetic causes of autonomous adrenal cortisol excess manifest with distinct steroid metabolomes.

### Autonomous adrenal mineralocorticoid excess

Autonomous aldosterone secretion [primary aldosteronism (PA)] is the most common disorder of adrenal steroidogenesis and represents the predominant cause of secondary hypertension, affecting at least 5% of the hypertensive population (156). The vast majority of PA cases correspond to two types: bilateral hyperaldosteronism (60% to 70%) and unilateral aldosterone-producing adenoma (APA; 30% to 40%) (156). Rare familial forms of PA have been shown to be associated with germline mutations in *KCNJ5* (157), *CACNA1D* (158), *CACNA1H* (159), and *CLCN2* (160). Similarly, somatic driver mutations in *KCNJ5*, *ATP1A1*, *ATP2B3*, *CACNA1D*, and *CTNNB1* have been discovered in APA tissue, altogether accounting for most cases (157, 158, 161–164). Most of these ion channel mutations lead to enhanced calcium influx into zona glomerulosa cells, thereby stimulating aldosterone synthase (CYP11B2) expression (165). Untreated PA exposes patients to a higher cardiovascular risk than for non-PA patients with similar degrees of hypertension (166–170). Differentiation between the different subtypes of PA is clinically important: unilateral disease is an indication for surgery whereas bilateral disease is best managed medically with MR blockers (156).

Biochemically, the hallmark of PA is the combination of high plasma aldosterone and suppressed renin (156). Some PA patients have high circulating levels of the two “hybrid” cortisol metabolites, 18-hydroxycortisol and 18-oxocortisol (Fig. 7) (115, 171, 172). Serum and urinary 18-hydroxycortisol, 18-oxocortisol, and 18-oxo-THF excretion are highest in FHA type 1 (= GRA, see “Glucocorticoid-remediable aldosteronism” above) (124, 173) and FHA type 3 (germline *KCNJ5* mutations) (113); they also tend to be higher in APA than in bilateral hyperaldosteronism, albeit with considerable overlap (115). No distinct steroid metabolome signature has been identified for other PA-causing mutations yet, mostly due to the rarity of cases.

Until recently, PA had been regarded as a disorder of mineralocorticoid biosynthesis only; however, it has now been shown that a large proportion of PA patients also demonstrate excessive urinary excretion of glucocorticoid metabolites, as well as of the 11-oxygenated androgen metabolite 11*β*-OHAn (148) (Fig. 8D). Both the conversions of 11-deoxycortisol to cortisol and A4 to 11OHA4 are catalyzed by the adrenal enzyme CYP11B1, and a recent study showed that the immunohistochemical expression of CYP11B1 in APA tissue correlated with the corresponding excretion of glucocorticoid and 11-oxygenated androgen metabolites (148). This prevalent cosecretion of both cortisol and aldosterone in PA, termed “Connshing syndrome” (148, 174), is likely to explain the reported increased risk of type 2 diabetes, osteoporosis, and depression in PA (153, 175–183), which have no intuitive link to mineralocorticoid activity, but represent commonly noted consequences of cortisol excess.

### Autonomous adrenal androgen excess

Adrenal androgen excess is a common feature of steroid excess in patients with ACC, although rarely isolated and more commonly cosecreted with other steroids. Isolated autonomous androgen excess from the adrenal gland can very rarely occur in the context of benign ACAs (1). Isolated macronodular hyperplasia of the zona reticularis is very rare, with only one case reported thus far (184), with the serum steroid profile revealing isolated overproduction of DHEA, DHEAS, and A4, unresponsive to dexamethasone-induced ACTH suppression.

### Mixed steroid excess: ACC

ACC is a rare but aggressive malignancy, which accounts for 2% to 11% of adrenal tumors (185). Prompt and accurate differentiation of ACC from benign ACA is the foremost clinical challenge posed by a new adrenal mass. The most useful radiological indicators of malignancy are size and lipid content: the likelihood of malignancy increases with size (186), and lipid-rich lesions are invariably benign (187). A considerable proportion of adrenal tumors, however, are difficult to classify by any imaging modality (187).

Routine serum biochemistry detects steroid hormone excess in 60% to 70% of ACC patients, with glucocorticoid and androgen excess dominating (1, 188); however, urine GC-MS profiling demonstrated steroid excess in >90% (1). Additionally, recent retrospective studies employing GC-MS–based urine steroid profiling revealed that ACCs present a unique steroidogenic “signature” characterized by accumulation of steroid hormone precursors (1, 154, 155) (Fig. 8E). The steroid biomarkers that are most helpful at distinguishing ACCs from ACAs include the glucocorticoid precursor metabolite THS, and the androgen precursor metabolites of Preg and 17OHPreg, 5PD, and 5PT (1). Multiple other Δ^5^ steroids are also excreted in excess. Recent preliminary retrospective studies also looked at the value of serum multisteroid profiling in detecting ACC (189, 190).

The combination of mass spectrometry–based steroid profiling with machine learning–driven analysis of the urinary steroid excretion data allowed for rapid detection of a “malignant steroid fingerprint” (Fig. 8E) (1) that can differentiate ACC from ACA with high sensitivity and specificity. A large-scale prospective test validation study has recently been completed and results are awaited; if positive, this could represent the first steroid metabolomics approach implemented as a routine diagnostic test.

Urine steroid metabolomics was also used to show that the detection of this malignant steroid fingerprint is not affected by concomitant mitotane treatment in ACC (191), suggesting a potential for noninvasive ACC follow-up monitoring. Additionally, urine steroid metabolome analysis revealed that mitotane strongly inhibits 5α-reductase activity in ACC patients, explaining treatment-related male hypogonadism, and acts as a strong inducer of CYP3A4, resulting in significantly accelerated cortisol inactivation. The latter explains the need for increased doses of glucocorticoid replacement therapy in patients with ACC receiving mitotane treatment (191).

## Outlook

The diagnostic potential of steroid metabolome analysis has been recognized since many decades ago and its application has now been extended from inborn steroidogenic disorders to autonomous adrenal steroid excess, yielding fascinating insights. In particular, comprehensive 24-hour urine steroid metabolome analysis has discovered novel steroid pathways and steroidogenic disorders. Recent progress in mass spectrometry technology and methodologies, combined with the development of customized computational approaches to facilitate urine steroid metabolomics analysis, is paving the way for more widespread use of mass spectrometry–based multisteroid profiling and steroid metabolomics approaches in clinical practice. The future is promising for steroid metabolomics, with likely widespread diagnostic and prognostic applications of this fascinating discovery tool.

## Additional Information

***Disclosure Summary:*** W.A. is an inventor on a patent for the use of steroid profiling as a biomarker tool in the differential diagnosis of steroid-producing and steroid-dependent tumors (PCT/GB2010/000274). The remaining authors have nothing to disclose.

***Data Availability:*** Data sharing is not applicable to this article as no datasets were generated or analyzed during the current study.

## Abbreviations

3*β*5*α*-PD: 5*α*-pregnane-3*β*,20*α*-diol
6*α*-OHTHA: 6*α*-hydroxy-11-dehydro-tetrahydrocorticosterone;
18OHB: 18-hydroxycorticosterone
A4: androstenedione
ACA: adrenocortical adenoma
ACC: adrenocortical carcinoma
ACRD: apparent cortisone reductase deficiency
AME: apparent mineralocorticoid excess
An: androgen
APA: aldosterone-producing adenoma
CAH: congenital adrenal hyperplasia
CMO I: corticosterone methyloxidase I
CMO II: corticosterone methyloxidase II
CRD: cortisone reductase deficiency
CYB5A: cytochrome b_5_
CYP: cytochrome P450
DOC: deoxycorticosterone
DSD: disorder of sex development
FHA: familial hyperaldosteronism
GC-MS: gas chromatography–mass spectrometry
GRA: glucocorticoid-remediable aldosteronism
hCG: human chorionic gonadotropin
HPA: hypothalamic–pituitary–adrenal
MACE: mild autonomous cortisol excess
MR: mineralocorticoid receptor
PA: primary aldosteronism
PCOS: polycystic ovary syndrome
PHA: pseudohypoaldosteronism
POR: cytochrome P450 oxidoreductase
PORD: POR deficiency
STS: steroid sulfatase
STSD: steroid sulfatase deficiency
T: testosterone
UHPLC-MS/MS: ultra-HPLC–tandem mass spectrometry
